# The WHO/ILO Joint Estimates approach to occupational risk factor and burden of disease estimation: providing actionable evidence with impact across sectors in countries

**DOI:** 10.1093/annweh/wxae107

**Published:** 2025-02-07

**Authors:** Tim Driscoll, Michelle C Turner, Paul J Villeneuve, Paul T J Scheepers, Vivi Schlünssen, Bochen Cao, Natalie C Momen, Frank Pega

**Affiliations:** Sydney School of Public Health, University of Sydney, NSW, 2006, Australia; Barcelona Institute for Global Health (ISGlobal), Doctor Aiguader 88, 08003 Barcelona, Spain; Department of Neuroscience, Carleton University, Health Sciences Building, Room 6307, 1125 Colonel By Drive, Ottawa, ON K1S 5B6, Canada; Radboud Institute for Biological and Environmental Sciences (RIBES), Radboud University, Heyendaalseweg 135, 6525 AJ Nijmegen, The Netherlands; Department of Public Health, Danish Ramazzini Centre, Aarhus University, Bartholins Allé 2, 8000 Aarhus, Denmark; Department of Data and Analytics, World Health Organization, 20 Avenue Appia, 1202 Geneva, Switzerland; Department of Environment, Climate Change and Health, World Health Organization, 20 Avenue Appia, 1202 Geneva, Switzerland; Department of Environment, Climate Change and Health, World Health Organization, 20 Avenue Appia, 1202 Geneva, Switzerland

## Background

Cherrie et al. (2024) identified challenges of approaches to estimating occupational risk factors (or exposures) and associated burden of disease and suggested solutions. They illustrated some points with comments on 2 products from the Joint Estimates of the World Health Organization (WHO) and the International Labour Organization (ILO) of the Work-related Burden of Disease and Injury (WHO/ILO Joint Estimates). Specifically, they offered new comments on the WHO/ILO Joint Estimates of non-melanoma skin cancer (NMSC) burden attributable to occupational exposure to solar ultraviolet radiation (UVR) (Pega et al. 2023c). They also repeated previous comments (Kromhout et al. 2023) on a WHO/ILO systematic review and meta-analysis on the prevalences and levels of occupational exposure to dusts and fibres (Schlünssen et al. 2023b), to which we have responded (Schlünssen et al. 2023a). We share Cherrie et al.’s (2024) interest to improve estimation approaches and acknowledge such approaches are continuously advancing (Pega et al. 2023b). Here, we outline how the WHO/ILO Joint Estimates approach addresses Cherrie *et al.*’s new comments (2024) and, beyond these, provides a global model. With this, we seek to contribute to the understanding and advancement of estimation approaches.

## The WHO/ILO Joint Estimates approach

Started in 2016 in the spirit of the Sustainable Development Goals, the WHO/ILO Joint Estimates are co-produced by the United Nations (UN) Specialized Agencies for Health and Labour that comprise over 193 Member States (WHO and ILO 2019). These estimates present the UN’s largest scientific collaboration on occupational health, with over 250 individual experts participating across 35 countries (Pega et al. 2023b; WHO 2023b). WHO and the ILO provide these estimates disaggregated from global to national level and by sex and age group. For many countries, these estimates may provide the only official occupational risk factor and disease burden information. These estimates enable individual and groups of countries and government sectors to prioritize, plan, cost, implement, monitor, and evaluate their workers’ health and safety laws, policies, and practices.

WHO and the ILO produce, approve, and report the WHO/ILO Joint Estimates under their strict organizational standards and procedures, fulfilling the *Fundamental Principles of Official Statistics* (UN General Assembly 2014). The organizations take several steps (Box 1) to apply their technical, statistical, quality assurance, ethics, and other standards and procedures to safeguard the quality, ethics, integrity, and independence of WHO/ILO Joint Estimates products. These steps comprise and go beyond those Cherrie et al. (2024) proposed and are informed by WHO’s and the ILO’s experiences estimating global disease burdens since the 1990s.

Applying the above approach (Box 1), WHO and the ILO, supported by partners, have provided several products from the WHO/ILO Joint Estimates:

- new methods for occupational risk factor (exposure) assessment and modelling (Pega et al. 2020b, 2022b, 2022c; Momen et al. 2022; Nafradi et al. 2022);
- pre-registered systematic reviews and meta-analyses of the prevalences, levels, and effects on health outcomes of prioritized occupational risk factors (Descatha et al. 2018, 2020; Godderis et al. 2018; Mandrioli *et al.*, 2018; Paulo et al. 2019; Rugulies et al. 2019, 2021; Teixeira et al. 2019, 2021a, 2021b; Tenkate et al. 2019; Li et al. 2018, 2020; Pega et al. 2020a, 2021a; Hulshof et al. 2019, 2021a, 2021b; Pachito et al. 2021; WHO 2021; Loomis et al. 2022; Schlünssen et al. 2023b);
- global, regional, and national estimates of occupational risk factors and/or burdens of disease for 43 risk factor-health outcome pairs for 183 countries (Pega et al. 2021b, 2022a, 2023c; WHO and ILO 2021a, 2021b);
- indicators for monitoring, policy, and practice (Pega et al. 2023a, 2024), launched and promoted with partners in China, Italy, Iran (Islamic Republic of), and South Africa; and
- an online application for visualising, downloading, and analysing estimates and indicators (WHO 2023a).

With these products, WHO and the ILO, together with partners, have substantially advanced the global understanding and monitoring of occupational risk factors and disease burden (Pega et al. 2023b). They demonstrated that, globally in 2016, an estimated 488 million working-age people were exposed to long working hours and that, of the estimated 1.9 million work-related deaths, this risk factor had the largest attributable number (0.8 million deaths; 40% of all work-related deaths) (WHO and ILO 2021a). The estimates also demonstrated that work-related *diseases* (not injuries) are the greatest burden (1.5 million deaths; 81% of work-related deaths) (WHO and ILO 2021a). The estimates have motivated public health, labour, and intersectoral actions to protect and promote workers’ health and safety in several countries. The estimates on long working hours (Pega et al. 2021b), for example, informed parliamentary debate and legislative and policy developments in Germany (German Parliament 2021), Japan (Ministry of Health Labour and Welfare 2023), Netherlands (Kingdom of the) (Ministry of Social Affairs and Employment 2023), and the United Kingdom of Great Britain and Northern Ireland (United Kingdom Parliament 2022), among other countries.

## Responses to specific comments by Cherrie et al. 2024

### Comparing burden of disease estimates

Cherrie et al. (2024) recommended comparing national estimates from global studies with those from national studies. They noted differences in national estimates of NMSC attributable to occupational exposure to UVR between the WHO/ILO Joint Estimates and selected national studies. As for all new WHO/ILO Joint Estimates, we reported and analysed these differences when presenting these estimates (see Table 1 in Pega et al. (2023c)). Prior to publication, WHO also consulted its 194 Member States (step 12 in Box 1), enabling countries to alert WHO/ILO of different national estimates with official recognition. We agree comparing estimates is sensible and note it helps identify what underlies differences.

Our analysis (Pega et al. 2023c) of differences in these occupational NMSC burden estimates between the WHO/ILO Joint Estimates and national studies (Young et al. 2012; Peters et al. 2019) differs from Cherrie et al.’s (2024). For Canada, Cherrie et al.’s (2024) extrapolation from Peters et al.’s (2019) number of occupational exposure-attributable incident cases to the number of occupational exposure-attributable deaths using a general population survival rate does not account for potential differences in survival by occupational exposure status; more precise extrapolation would require a comprehensive and transparent evaluation of the relevant body of evidence (see Stevens et al. (2016)). For the United Kingdom, both the WHO/ILO Joint Estimates and Young *et al.*’s estimates (2012) of the number of occupational exposure-attributable NMSC deaths indicate that this burden is only small. When comparing national estimates by latitude (Cherrie et al. 2024), factors such as exposure prevalence, risk ratio, and population size play key roles in estimating the occupational exposure-attributable fraction, and the WHO/ILO Joint Estimates of the occupational exposure-attributable NMSC burdens are calculated using WHO Global Health Estimates of the total envelopes of NMSC burdens that already reflect differences by latitude (Step 8 in Box 1) (WHO 2020).

Additionally, producers of estimates must strive for accuracy, but unless reporting systems are complete, the precise number of non-fatal or fatal disease events caused by an occupational risk factor will never be known. Global estimation allows between-country comparisons using consistent data sources and methods but may not necessarily produce the most accurate estimate for each country. If the focus is preventing diseases caused by occupational risk factors, burden of disease estimates can contribute information on whether their attributable burdens of disease are large enough to warrant policy and action, and their comparative burdens. Since assumptions, included risk factors, and included health endpoints may differ, estimates are expected to differ (Driscoll et al. 2005; Coggon 2022).

Cherrie et al. (2024) also suggested expanding the scope of the occupational comparative risk assessment by adding ‘agents that are probable causes of diseases concerned, not where causation is unequivocal’ (p. 676). Arguments exist for and against more inclusive or exclusive criteria, but standardization is more likely when only risk factor-health outcome pairs with strong evidence of a causal relationship are included. Accordingly, WHO/ILO Joint Estimates are only produced for pairs reaching a WHO/ILO strength of evidence rating of “sufficient evidence for harmfulness” (step 5 in Box 1) (Pega et al. 2021a).

### Using risk ratios on non-fatal event to estimate burden of fatal event

Cherrie et al. (2024) questioned WHO and the ILO’s use of a risk ratio for non-fatal NMSC to estimate the burden of fatal NMSC in Pega et al. (2023c). The organizations, supported by individual experts, conducted a pre-registered systematic review and meta-analysis on the effect of occupational exposure to UVR on NMSC non-fatal (incident) event and fatal event (WHO 2021) (steps 3 and 4 in Box 1). Strength of evidence assessment ratings were ‘sufficient’ and ‘inadequate’ evidence for harmfulness for NMSC incident event and fatal event, respectively (WHO 2021). The organizations applied their pre-specified criteria, selecting as the ‘best’ effect estimate the risk ratio on incident event (Pega et al. 2023c). This selection should only introduce bias if the severity (rather than just the risk) of the resulting disease varies with exposure level. These criteria have been applied across WHO/ILO Joint Estimates (Pega et al. 2021b, 2023c), previous WHO burden of disease estimations (Concha-Barrientos et al. 2004), and Global Burden of Disease Studies (GBD Risk Factors Collaborators 2024).

### Avoiding data with potential interests

Cherrie et al. (2024) suggested the use of ‘non-peer-reviewed data from […] employers, unions’ (p. 676). For official and other estimates, using data from producers with financial or other interests (whether perceived or actual) risks loss of trustworthiness. Independence and ethics are inherent to the WHO/ILO Joint Estimates and other official statistics (UN General Assembly 2014). Strict procedures exist to manage declarations of interests of individual experts seeking to contribute to the WHO/ILO Joint Estimates (step 2 in Box 1). Official and other estimates must be safeguarded against conflicting interests to protect their technical and ethical integrity and trustworthiness.

Box 1 **: WHO/ILO Joint Estimates approach to occupational risk factor and burden of disease estimation**

1. Based on pre-specified criteria, systematically prioritize a pair of occupational risk factor and health outcome for systematic review and meta-analysis of the latest global evidence base.
2. Establish a Technical Advisory Group of individual experts on the prioritized risk factor-health outcome pair through issuing an open call for individual experts, managing declarations of interest of applicants, and systematically selecting a group of individual experts representative across WHO Regions and sexes.
3. Supported by the Technical Advisory Group, produce a protocol for the systematic review and meta-analysis on the risk factor-health outcome pair that applies the standard WHO/ILO systematic review methods (Pega et al. 2021a) and publish it in a peer-reviewed, open-access publication.
4. Supported by the Technical Advisory Group, produce the systematic review and meta-analysis on the risk factor-health outcome pair and publish these in a peer-reviewed, open-access publication.
5. Based on the systematic review and meta-analysis, judge if there is ‘sufficient evidence’ to proceed to estimation of the exposure to the occupational risk factor and of the burden of disease attributable to the risk factor.
6. If there is ‘sufficient evidence’, use standard WHO/ILO data and methods (Pega et al. 2021b, 2023c; WHO and ILO 2021a) to combine the relevant WHO/ILO joint database of exposure to the risk factor with the risk measures produced in the systematic review and meta-analysis.
7. Consult and coordinate within and between WHO and the ILO on the data sources, methods, and estimates produced.
8. Use WHO Global Health Estimates of burden of disease (which themselves applied a rigorous methodology to generate point estimates and uncertainty intervals and have undergone a thorough consultation process with Member States; WHO (2020)) to ensure consistency and comparability across the WHO/ILO Joint Estimates and of the WHO/ILO Joint Estimates with other estimates in the WHO Comparative Risk Assessment.
9. Produce primary models and estimates of exposure to the risk factor and, subsequently, of the burden of disease from the health outcome that is attributable to the risk factor.
10. Produce sensitivity analyses to investigate the impact of changes in assumptions or input variables or their values on the estimates and report their results.
11. Model uncertainty across input parameters and produce and report uncertainty intervals for the risk factor and burden of disease estimates.
12. Consult the 194 Member States of WHO through an official WHO country consultation of representatives in Ministries of Health and Permanent Missions, with outreach to Ministries of Labour in countries.
13. Consult leading national technical agencies (eg national institutes of occupational health and safety), individual and groups of experts, and peer-reviewers.
14. Use feedback received in country and other consultations to further improve the estimation and estimates of exposure to the risk factor and the attributable burden of disease (as is feasible).
15. Undergo WHO and ILO statistical reviews and organizational clearances (authorizations) of the estimates of exposure to the risk factor and the attributable burden of disease as official WHO health estimates and official ILO estimates.
16. Publish the estimates of exposure to the risk factor and the attributable burden of disease and report them in compliance with the *GATHER Guidelines for Accurate and Transparent Health Estimates Reporting* (Stevens et al. 2016) in a peer-reviewed, open-access publication.
17. Add the estimates of exposure to the risk factor and the attributable burden of disease in the open-access WHO Occupational Burden of Disease Application (WHO 2023a) for data visualisation, download, and health inequality analysis.
18. Disseminate the estimates of exposure to the risk factor and the attributable burden of disease through a global launch targeting government representatives, communities of affected workers, and the public (eg researchers).
19. Update and (if feasible) improve the estimates of exposure to the risk factor and the attributable burden of disease and report them on a regular cycle as part of the broader WHO and WHO/ILO Joint Comparative Risk Assessments.

WHO/ILO Joint Estimates are produced wherever feasible from exposure data collected by national statistical offices. The estimates of NMSC burden attributable to occupational exposure to UVR, for example, used 166 million official data points on occupation from 763 Labour Force Surveys collected by 96 countries between 1996 and 2021 (Pega et al. 2023c). This use of official exposure data strengthens governments’ ownership of and trust in the WHO/ILO Joint Estimates and enables them to reproduce their national estimates. WHO’s open-access policies for data (where permitted), estimates, and publications make its estimation and estimates transparent and publicly available to all users.

## Conclusions

The WHO/ILO Joint Estimates follow comprehensive and transparent technical, statistical and ethics standards, assuring high quality and safeguarding ethics and independence. This approach has established these unique UN interagency estimates as a trusted source of high-quality information with an impact on workers’ health and safety policy and practice across health and labour sectors in countries. It presents a feasible estimation model, at least for sizeable governmental or intergovernmental organizations with the required time, funds, organizational structures (eg independent clearance function), and procedures (eg quality assurance assessments).

We agree with many of Cherrie *et al.*’s new comments (2024), but see value in applying different methods, with different assumptions, and expect these would result in differences in estimates. Critical evaluation of these differences provides insight for improving estimation methods. Enhanced and standardized reporting of estimates (Stevens et al. 2016) will strengthen between-study comparisons. We consider the established standard of prioritizing strong risk ratios on non-fatal event to estimate burden of fatal event sensible when strong risk ratios for fatal event are unavailable. Estimates (especially official ones) must avoid input data with actual or perceived conflict of interests.

Burden of disease estimation seeks to identify and highlight risk factors that are significant causes of occupational burden of disease. The resulting estimates can guide policy and decision makers to prioritize these risk factors and health outcomes for exposure and disease or injury prevention and control. This evidence can, ultimately, be used to protect and promote workers’ health and safety in policy and practice in countries.

## Data Availability

No data were used in this study.
